# Immune‐mediated axonal dysfunction in seropositive and seronegative primary Sjögren’s syndrome

**DOI:** 10.1002/acn3.51053

**Published:** 2020-05-15

**Authors:** Jowy Tani, Hsien‐Tzung Liao, Hui‐Ching Hsu, Lung‐Fang Chen, Tsui‐San Chang, Cindy Shin‐Yi Lin, Jia‐Ying Sung

**Affiliations:** ^1^ Department of Neurology Wan Fang Hospital Taipei Medical University Taipei Taiwan; ^2^ Ph.D. Program for Neural Regenerative Medicine College of Medical Science and Technology Taipei Medical University and National Health Research Institutes Taipei Taiwan; ^3^ Division of Allergy, Immunology and Rheumatology Department of Internal Medicine Taipei Veterans General Hospital Taipei Taiwan; ^4^ School of Medicine College of Medicine National Yang‐Ming University Taipei Taiwan; ^5^ Division of Allergy, Immunology and Rheumatology Department of Internal Medicine Wan Fang Hospital Taipei Medical University Taipei Taiwan; ^6^ Division of Allergy, Immunology and Rheumatology Department of Internal Medicine School of Medicine College of Medicine Taipei Medical University Taipei Taiwan; ^7^ Neural Regenerative Medicine College of Medical Science and Technology Taipei Medical University and National Health Research Institutes Taipei Taiwan; ^8^ Central Clinical School Faculty of Medicine and Health Brain & Mind Centre The University of Sydney Sydney Australia; ^9^ Department of Neurology School of Medicine College of Medicine Taipei Medical University Taipei Taiwan

## Abstract

**Objective:**

The present study investigates the peripheral neuropathy in Primary Sjögren's syndrome (pSS) using the nerve excitability test to further elucidate how peripheral nerves are affected by the autoantibodies.

**Methods:**

Each patient received clinical evaluation, examination for anti‐SSA/Ro and anti‐SSB/La antibodies titer, paired motor and sensory nerve excitability test, thermal quantitative sensory test (QST), and nerve conduction study (NCS).

**Results:**

A total of 40 pSS patients wasenrolled. Motor axonal study of the pSS with positive anti‐SSA/Ro or anti‐SSB/La antibodies (*n* = 28) was found to have increased stimulus for 50% compound muscle action potential (CMAP) (*P* < 0.05), increased rheobase (*P* < 0.01), increased minimum I/V slope (*P* < 0.01) and hyperpolarizing I/V slope (*P* < 0.05), increased relative refractory period (RRP, *P* < 0.001), decreased accommodation of threshold electrotonus toward depolarizing current (*P* < 0.05), and increased accommodation toward hyperpolarizing current (*P* < 0.05). Seronegative pSS (*n* = 10) showed much less prominent motor axonal changes, showing only increased minimum I/V slope (*P* < 0.05). Sensory axonal study in seropositive pSS patients is found to have increased stimulus for 50% sensory nerve action potential (SNAP) (*P* < 0.01), decreased latency (*P* < 0.01), increased RRP (*P* < 0.01), and increased subexcitability (*P* < 0.05). Seronegative pSS patients have shown no significant sensory axonal changes. Thermal QST showed more prominent abnormalities in seronegative pSS compared to seropositive pSS.

**Interpretation:**

Anti‐SSA/Ro and anti‐SSB/La autoantibodies might cause dysfunction in nodal and internodal region of the axon and small nerve fibers; meanwhile, autoreactive antibodies in seronegative pSS mainly affect small nerve fibers. Thus, the underlying pathophysiology for the two types of pSS is different.

## Introduction

Primary Sjögren's syndrome (pSS) is an autoimmune disease that affects both East and West.^1^ To date, more than a dozen autoreactive antibodies have been detected in pSS patients.^2^ The major antibodies in pSS are autoantibodies against SSA/Ro and SSB/La autoantigens, and these represent a diagnostic tool for the disease.^3^ The autoantibodies might lead to the destruction of epithelial cells, causing gland hypofunction and the development of sicca symptoms, and these autoantibodies would also lead to extraglandular manifestations, affecting cutaneous, articular, pulmonary, cardiovascular, nephro‐urological, nervous, and hematological systems.^4^, ^5^, ^6^

Among its many systemic manifestations, peripheral neuropathy in pSS significantly lowered the patients’ quality of life.^7^, ^8^ The majority of studies reported a prevalence of peripheral neuropathy occurs alongside pSS in about 5–15% of cases.^9^, ^10^, ^11^

In one study, sensory peripheral neuropathy was found to be associated with the presence of anti‐SSA and anti‐SSB antibodies.^12^ Nevertheless, overall we still have limited knowledge of how the autoantibodies affect the peripheral nervous system. Peripheral neuropathy in pSS tended to be underestimated due to the lack of a sensitive and noninvasive diagnostic tool, and there is little data available on how the electrophysiologic properties of peripheral nerves are affected.

In this study, we would utilize the motor and sensory nerve excitability test to assess axonal membrane properties of pSS patients noninvasively.^13^ The present study would attempt to identify axonal ion channels and pump dysfunction in pSS, to unveil the possible roles of anti‐SSA and anti‐SSB antibodies in its pathogenesis, and to explore the potential of the nerve excitability test in the evaluation of peripheral neuropathy in pSS.

## Methods

### Participants

Patients meeting 2002 AECG criteria for pSS^3^ and at least 18 years old were enrolled in the pSS group of the study. The exclusion criteria for the study are diabetes mellitus, renal failure, and other metabolic diseases known to affect nerve excitability, such as vitamin B12 deficiency, alcohol abuse, uremia.

The enrolled pSS patients were categorized into the seropositive group if a patient tested positive on either anti‐SSA or anti‐SSB, or into the seronegative group if tested negative on both anti‐SSA and anti‐SSB.

**Figure 1:**
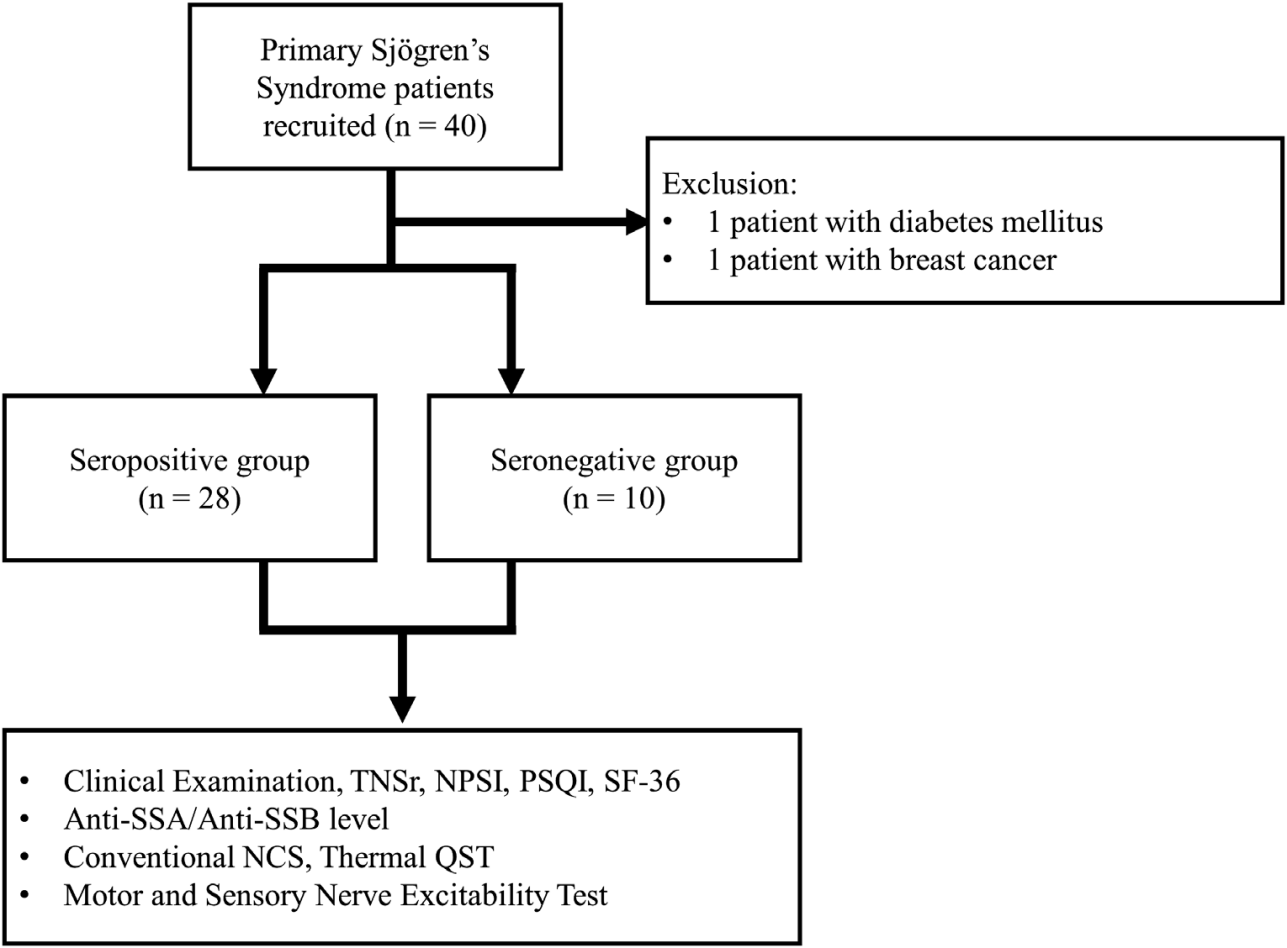
Flow chart of the present study. Patients are categorized into the seropositive and seronegative group based on Anti‐Ro/SSA and Anti‐La/SSB status. NPSI, Neuropathic pain scale inventory; PSQI, Pittsburg sleep quality index; SF‐36, 36‐item short form; TNSr, Total neuropathy score‐reduced.

In addition, sex and age‐matched healthy participants are also enrolled in the control group of the study, to act as healthy controls (mean age 66.32 ± 1.44 years, *n* = 10). Flow chart of the present study is shown in Figure 1.

### Clinical, laboratory, and conventional electrophysiologic evaluation

Each patient underwent detailed history taking and physical examination (including standard neurological examination). During the clinical evaluation, Total neuropathy score‐reduced (TNSr),^14^ Neuropathic pain scale inventory (NPSI),^15^ Pittsburg sleep quality index (PSQI),^16^ and 36‐item short form (SF‐36) health survey^17^ for assessment of the quality of life were also obtained. In particular, Physical Component Summary (PCS) and Mental Component Summary (MCS) for each patient are summarized based on the SF‐36 survey result.

Examination for anti‐SSA and anti‐SSB antibodies titer was performed on each pSS patient. Routine blood tests and serum creatinine levels were also obtained.

Each patient received conventional nerve conduction study (NCS) and thermal quantitative sensory test (QST). Conventional NCS assessing median, ulnar, peroneal, tibial, and sural nerves, and were performed in all patients using standard clinical neurophysiology equipment. Cold and warm threshold of upper and lower limbs were also obtained for each patient using thermal QST. The presence of peripheral neuropathy according to conventional NCS and thermal QST was determined for each patient.^18^, ^19^, ^20^

### Nerve excitability testing

The paired recording of motor and sensory nerve excitability studies were performed by stimulating the nerve median at the wrist according to previously described protocols. Skin temperature over the wrist was maintained at greater than or equal to 32.0 degree Celsius.^13^, ^21^ Motor and sensory nerve excitability indices were obtained for each subject. Compound muscle action potentials (CMAPs) were recorded from the abductor pollicis brevis muscle, and sensory nerve action potentials (SNAPs) were recorded from the index finger Stimulation and recording were manipulated by software (QTRAC version 28/10/2011; Institute of Neurology, London, U.K.) and stimulus current was administered using an isolated linear bipolar constant‐current stimulator (DS5; Digitimer, Welwyn Garden City, U.K.). The changes in current required to produce a target potential corresponding to 50% of the maximal CMAP or SNAP were tracked. Latency was defined as the time delay (ms) between stimulus onset and peak CMAP or SNAP response. The stimulus threshold was defined as the current (mA) necessary to produce amplitudes of CMAP or SNAP response of half‐maximal amplitude.

The nerve excitability protocol incorporated the following recordings: (1) a stimulus‐response (SR) curve; (2) strength‐duration (SD) relationship that determined rheobase and strength‐duration time constant (SDTC); (3) threshold electrotonus (TE) utilizing subthreshold 100‐ms polarizing currents in both depolarizing (TEd; +40%) and hyperpolarizing (TEh; −40%) directions to alter the potential difference across the internodal membrane; (4) recovery cycle (RC) using a paired‐pulse paradigm with a supramaximal conditioning stimulus followed by a test stimulus at interstimulus intervals from 2 to 200 ms. Superexcitability was measured as the maximal threshold reduction and subexcitability as the maximal threshold increase after an interstimulus interval of 10 ms; and (5) current‐threshold (I/V) relationship was obtained by measuring threshold changes following subthreshold polarizing currents of 200 ms duration, with current strength gradually altered in 10% steps from 50% (depolarizing) to 100% (hyperpolarizing). Abnormalities in nerve excitability parameters detected by the nerve excitability test were considered as axonal dysfunction.

### Statistical analysis

Nerve excitability recording data of seropositive pSS patients, seronegative pSS patients, and healthy controls were compared with unpaired T‐tests. Equality of variances was calculated with Levene’s test. To determine the effect of autoantibodies on the nerve excitability, correlation studies were performed with Pearson R or Spearman’s Rho.

Data analysis was performed using the QTRAC software and Statistical Package for the Social Sciences (SPSS) for windows version 21 (SPSS Inc., Chicago, USA).

All data are presented as mean ± standard error of the mean. P values were considered significant if ≤0.05.

### Study approval and patient consent

All patients enrolled in the study are recruited from Wan Fang Hospital, Taipei Medical University, Taipei, Taiwan. The present study was approved by the Joint Institution Review Board of Taipei Medical University. All the subjects gave their signed informed consent for inclusion in the present study.

### Data availability

Anonymized data not published within the article will be shared by request from any qualified investigator.

## Results

### Patient clinical and laboratory profiles

To date, we have obtained adequate motor and sensory nerve excitability testing from 40 pSS patients. Of these, one patient was excluded for diabetes mellitus, and one patient for breast cancer. Twenty‐eight patients are in the seropositive group, and 10 patients are in the seronegative group.

The demographics and clinical profiles for the patients are shown in Table 1. All pSS patients and healthy controls enrolled in the present study were women. The age for seropositive patients is 59.07 ± 2.38 years, and 62.10 ± 2.68 years for seronegative patients. The anti‐SSA level was 111.81±.18.14 U/mL in seropositive patients and 0.42 ± 0.12 U/mL in seronegative patients. The anti‐SSB level was 41.27±.18.95 U/mL in seropositive patients and 0.28 ± 0.06 U/mL in seronegative patients.

**Table 1:** Patient demographics and clinical profiles.

Variable	Primary Sjögren’s Syndrome (n = 38)	
	Seropositive (n = 28)	Seronegative (n = 10)
Age (yr.)	59.00 (2.37)	62.10 (2.68)
Anti‐Ro/SSA titer (U/ml)	111.81 (18.14)**	0.42 (0.12)
Anti‐La/SSB titer (U/ml)	80.62 (16.04)*, 1	0.28 (0.06)
Dry‐eyes duration (months)	80.62 (16.04)	96.70 (25.80)
Dry‐mouth duration (months)	74.92 (11.23)	93.60 (22.30)
Peripheral nerve symptoms duration (months)	57.12 (12.16)	43.90 (12.70)
Clinical findings		
Weakness (%)	7	10
Abnormal pain/temperature sensation (%)	85.71	80
Abnormal vibratory sensation/proprioception (%)	35.71	40
Autonomic dysfunction (%)	35.71	40
Peripheral neuropathy		
Small fiber neuropathy by thermal QST only (%)	21.42	30
Neuropathy by conventional NCS only (%)	10.71	20
Small fiber neuropathy + neuropathy (%)	39.29	30
TNSr (score)	5.27 (0.60)	5.90 (0.64)
NPSI (score)	6.77 (1.29)	7.15 (2.19)
Maximal pain score	4.31 (0.47)	4.90 (0.50)
PSQI (score)	9.00 (0.86)	7.93 (1.29)
SF‐36 PCS (score)	45.64 (1.75)	49.33 (2.88)
SF‐36 MCS (score)	44.81 (2.10)	43.80 (2.71)

The reported values of age, symptoms duration, laboratory data, and clinical scoring represent mean (standard error). The reported values of abnormal clinical findings represent percentage. TNSr, Total neuropathy score‐reduced ^14^; NPSI, Neuropathic pain symptom inventory ^15^; PSQI, Pittsburg sleep quality index ^16^; SF‐36, 36‐item short form ^17^; PCS, physical component summary; MCS, mental component summary.

* *P* < 0.05 versus seronegative.

** *P* < 0.01 versus seronegative.

There are 7% of seropositive and 10% of seronegative pSS patients with weakness symptoms. Also, 53.5% of seropositive and 90% of seronegative patients had hypo/hyperreflexia. Meanwhile, 85.71% of seropositive and 80% of seronegative patients were found to have abnormal pain/temperature sensation, and 35.71% of seropositive and 40% of seronegative patients were found to have abnormal vibratory sensation/proprioception.

Also, 21.43% of seropositive and 30% of seronegative patients had small fiber neuropathy evidenced on QST examination only, while 10.71% of seropositive and 20% of seronegative patients had peripheral neuropathy evidenced on conventional NCS only. There are 39.29% of seropositive and 20% of seronegative patients that had abnormalities on both thermal QST and NCS.

The TNSr for seropositive and seronegative patients were 5.27 ± 0.60 and 5.90 ± 0.64, NPSI were 6.77 ± 1.29 and 7.15 ± 2.19, and PSQI were 9.00 ± 0.86 and 7.93 ± 1.29, respectively. The SF‐36 revealed a PCS of 44.94 ± 8.69 and MCS of 46.43 ± 10.78 for seropositive, and PCS of 50.88 ± 9.34 and MCS of 45.28 ± 8.97 for seronegative patients.

Summary of conventional NCS and thermal QST results is shown in Table 2. For seropositive patients, Z‐scores for upper limb warm threshold was 1.54 ± 3.13, upper limb cold threshold was −0.60 ± 2.83, lower limb warm threshold was 1.48 ± 1.37, lower limb cold threshold was −2.06 ± 4.18. For seronegative patients, Z‐scores for upper limb warm threshold was 2.77 ± 3.58, upper limb cold threshold was −1.18 ± 2.67, lower limb warm threshold was 1.52 ± 1.34, lower limb cold threshold was −2.44 ± 1.56 (normal Z score range for this laboratory is between −2 and 2).

**Table 2:** Conventional NCS and thermal QST results summary.

Variable	Primary Sjögren’s Syndrome (*n* = 38)	
	Seropositive (*n* = 28)	Seronegative (*n* = 10)
Median motor NCV (m/s)	54.12 (1.00)	55.67 (1.37)
Median motor amplitude (mV)	7.92 (0.59)	7.69 (0.57)
Median sensory NCV (m/s)	55.33 (1.41)	57.67 (2.64)
Median sensory amplitude (μV)	42.92 (3.72)	33.33 (3.01)
Upper limb warm threshold (Z score)	2.38 (1.05)^*^, ^1^	4.54 (2.24)^*^, ^1^
Upper limb cold threshold (Z score)	−1.06 (0.72)	−2.19 (1.34)^*^, ^1^
Lower limb warm threshold (Z score)	1.56 (0.27)	1.82 (0.43)
Lower limb cold threshold (Z score)	−2.16 (0.80)^*^, ^1^	−3.14 (0.64)^*^, ^1^

The reported values of laboratory data represent mean (standard deviation). NCV, nerve conduction velocities; QST, quantitative sensory testing.

^1^ Mean data is out of normal range for this laboratory. The normal ranges in this laboratory were: median motor NCV 49.2–64.8 m/s, median motor amplitude 3.0–15.4 mV, median sensory NCV 48.7–65.5 m/s, median sensory amplitude 10.0–72.6 μV, Z score between −2 and 2.

### Motor axonal dysfunction in seropositive and seronegative pSS

**Figure 2:**
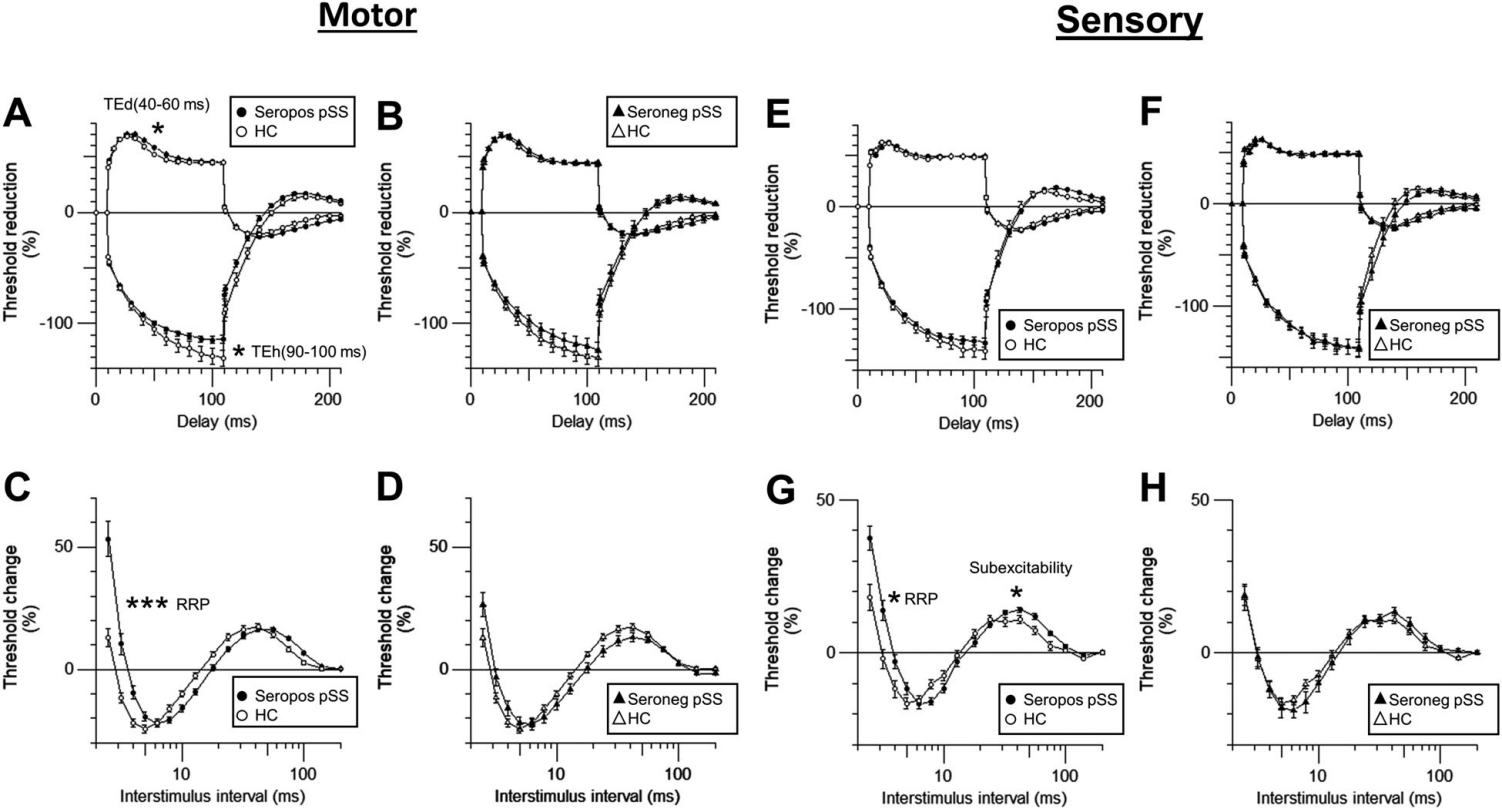
Motor and sensory axonal excitability test of healthy control and pSS. (A and B) Comparison of stimulus‐response curve, (C and D) strength‐duration time constant, (E and F) recovery cycle, and (G and H) threshold electrotonus (Healthy control: line, seropositive pSS: filled circle, and seronegative pSS: empty circle). Motor profiles are shown in the left column, while sensory are shown in the right.

Motor nerve excitability parameters of healthy controls, seropositive, and seronegative pSS patients are shown in Table 3 and Figure 2A‐D. Seropositive patients had significantly increased stimulus for 50% CMAP (*P* < 0.05), increased rheobase (*P* < 0.01), increased minimum I/V slope (*P* < 0.01) and hyperpolarizing I/V slope (*P* < 0.05). There was also increased relative refractory period (RRP, *P* < 0.001), increased refractoriness at 2.5 ms (*P* < 0.001) and increased accommodation of TE toward depolarizing current (TEh[40–60 ms], *P* < 0.05) and hyperpolarizing current (TEh[90–100 ms], *P* < 0.05).

**Table 3:** Comparison of nerve excitability parameters between groups.

Axonal properties	Healthy Controls (*n* = 10)	Primary Sjögren’s syndrome patients (*n* = 38)			
		Seropositive (*n* = 28)	*P* value	Seronegative (*n* = 10)	*P* value
Motor stimulus‐response curve					
Stimulus for 50% CMAP (mA)	2.53 ± 0.25	3.54 ± 0.25	<0.05	3.02 ± 0.56	NS
Peak response (mV)	8.11 ± 0.76	7.53 ± 0.45	NS	7.51 ± 0.79	NS
Latency (ms)	6.32 ± 0.31	6.43 ± 0.15	NS	6.27 ± 0.3	NS
Rheobase (mA)	1.68 ± 0.16	2.26 ± 0.17	<0.01	1.91 ± 0.4	NS
Motor SDTC (ms)	0.42 ± 0.01	0.51 ± 0.02	NS	0.55 ± 0.06	NS
Motor I/V relationship					
Resting I/V slope	0.59 ± 0.04	0.61 ± 0.02	NS	0.58 ± 0.04	NS	
Minimum I/V slope	0.22 ± 0.01	0.28 ± 0.01	<0.01	0.26 ± 0.02	<0.05
Hyperpolarizing I/V slope	0.34 ± 0.02	0.45 ± 0.03	<0.05	0.54 ± 0.08	NS
Motor recovery cycle					
RRP	2.86 ± 0.06	3.61 ± 0.14	<0.001	3.2 ± 0.17	NS
Refractoriness at 2.5 ms	12.96 ± 3.56	53.39 ± 7.16	<0.001	26.53 ± 4.85	<0.05
Superexcitability (%)	−22.79 ± 1.6	‐22.09 ± 1.09	NS	−21.85 ± 2.25	NS
Superexcitability at 5 ms	−24.44 ± 1.59	‐19.29 ± 1.83	NS	−21.76 ± 2.5	NS
Subexcitability (%)	16.3 ± 1.44	16.01 ± 0.85	NS	12.71 ± 1.72	NS
Motor threshold electrotonus					
TEd(40‐60 ms) (%)	48.1 ± 1.24	52.88 ± 0.96	<0.05	50.11 ± 1.64	NS
TEd(peak) (%)	66.32 ± 1.44	68.84 ± 0.84	NS	67.36 ± 2.07	NS
TEh(90‐100 ms) (%)	−130.17 ± 7.22	−114.32 ± 3.24	<0.05	−121.63 ± 7.29	NS
Sensory stimulus‐response curve					
Stimulus for 50% SNAP (mA)	1.75 ± 0.22	3.15 ± 0.23	<0.001	2.33 ± 0.33	NS
Peak response (μV)	36.82 ± 3.87	43.85 ± 3.26	NS	36.73 ± 3.94	NS
Latency (ms)	3.27 ± 0.12	3.25 ± 0.08	<0.001	2.99 ± 0.14	NS
Rheobase	0.72 ± 0.08	1.44 ± 0.12	NS	0.98 ± 0.16	NS
Sensory SDTC (ms)	0.6 ± 0.03	0.56 ± 0.02	NS	0.64 ± 0.11	NS
Sensory I/V relationship					
Resting I/V slope	0.58 ± 0.04	0.53 ± 0.01	NS	0.63 ± 0.1	NS
Minimum I/V slope	0.22 ± 0.01	0.23 ± 0.01	NS	0.21 ± 0.01	NS
Hyperpolarizing I/V slope	0.36 ± 0.06	0.34 ± 0.02	NS	0.35 ± 0.03	NS
Sensory recovery cycle					
RRP	3.17 ± 0.16	3.91 ± 0.16	<0.05	3.18 ± 0.15	NS
Refractoriness at 2.5 ms	18.2 ± 4.15	37.61 ± 3.91	<0.01	18.7 ± 3.22	NS
Superexcitability (%)	−15.51 ± 1.55	‐16.15 ± 1.29	NS	−18.21 ± 2.71	NS
Subexcitability (%)	10.93 ± 1.12	13.86 ± 0.63	<0.05	12.27 ± 1.35	NS
Sensory threshold electrotonus					
TEd(peak) (%)	60.11 ± 0.67	59.81 ± 0.75	NS	59.5 ± 1.26	NS
TEh(10‐20 ms) (%)	−87.82 ± 2.05	−84.58 ± 1.33	NS	−85.31 ± 2.46	NS
TEh(20‐40 ms) (%)	−109.33 ± 3.61	‐105.42 ± 2.07	NS	−107.43 ± 3.4	NS
TEh(90‐100 ms) (%)	−140.43 ± 7.98	−132.79 ± 4	NS	−141.53 ± 7.29	NS

The reported values represent mean ± standard error and the *P*‐value from unpaired T‐test with healthy controls.

SNAP, sensory nerve action potential; CMAP, compound muscle action potential; SDTC, strength‐duration time constant; RRP, relative refractory period; NS, not statistically significant.

Meanwhile, seronegative pSS patients showed somewhat different motor axonal changes pattern, showing only increased minimum I/V slope (*P* < 0.05) and refractoriness at 2.5 ms (*P* < 0.05). No significant axonal changes are observed in either SR curve or TE.

### Sensory axonal dysfunction in seropositive and seronegative pSS

Sensory axonal excitability indices of healthy controls, seropositive, and seronegative pSS patients are also shown in Table 3 and Figure 2E–H. Seropositive patients are found to have increased stimulus for 50% SNAP (*P* < 0.01), increased patency (*P* < 0.01), increased RRP (*P* < 0.05), increased refractoriness at 2.5 ms (*P* < 0.01), and increased subexcitability (*P* < 0.05).

Seronegative pSS patients have shown no significant axonal changes are observed in either SR curve, I/V relationship, TE, or RC.

### Correlation studies between clinical parameters and excitability parameters

In motor axonal study of seropositive patients, anti‐SSA level is correlated with hyperpolarized I/V slope (Rho=−0.46, *P* < 0.05), while duration of dry eyes was correlated with SDTC (Rho = 0.45, *P* < 0.05), and superexcitability (*R* = 0.41, *P* < 0.05); duration of dry mouth was correlated with TEd(40–60 ms) (*R* = 0.40, *P* < 0.05).

In sensory axonal study of seropositive patients, anti‐SSA level was correlated with stimulus for 50% SNAP (*R* = 0.41, *P* < 0.05), TEd(undershoot) (*R* = −0.44, *P* < 0.05), and TEh(overshoot) (*R* = 0.71, *P* < 0.01). Anti‐SSB level was correlated with TEh(overshoot) (Rho = 0.44, *P* < 0.05). Dry eyes duration was correlated with RRP (Rho = 0.45, *P* < 0.05), TEd(10–20 ms) (Rho = −0.39, *P* < 0.05), Superexcitability at 5 ms (Rho = 0.46, *P* < 0.05). Duration of neurological symptoms was correlated with minimum I/V slope (Rho = 0.41, *P* < 0.05), resting I/V slope (Rho = 0.55, *P* < 0.01), TEh(90–100 ms) (Rho = 0.48, *P* < 0.05). TNSr was correlated with hyperpolarized I/V slope (*R* = 0.47, *P* < 0.05) and superexcitability at 5 ms (*R* = 0.41, *P* < 0.05). NPSI level was correlated with the minimum I/V slope (Rho = 0.44, *P* < 0.05). SF‐36 MCS was correlated with subexcitability (*R* = 0.43, *P* < 0.05), and TEh(overshoot) (*R* = 0.39, *P* < 0.05).

In the motor axonal study of seronegative patients, dry mouth duration was correlated with peak response (*R* = 0.63, *P* < 0.05), TEh(90–100 ms) (*R* = 0.82, *P* < 0.01), TEd(10–20 ms) (*R* = −0.68, *P* < 0.05).

In the sensory axonal study of seronegative patients, the duration of neurological symptoms was correlated with subexcitability (*R* = −0.70, *P* < 0.05). TNSr was correlated with the hyperpolarized I/V slope (Rho = 0.89, *P* < 0.01). The pain score was correlated to TEh(overshoot) (*R* = 0.66, *P* < 0.05), and NPSI was correlated to resting I/V slope (Rho = 0.65, *P* < 0.05).

## Discussion

**Figure 3:**
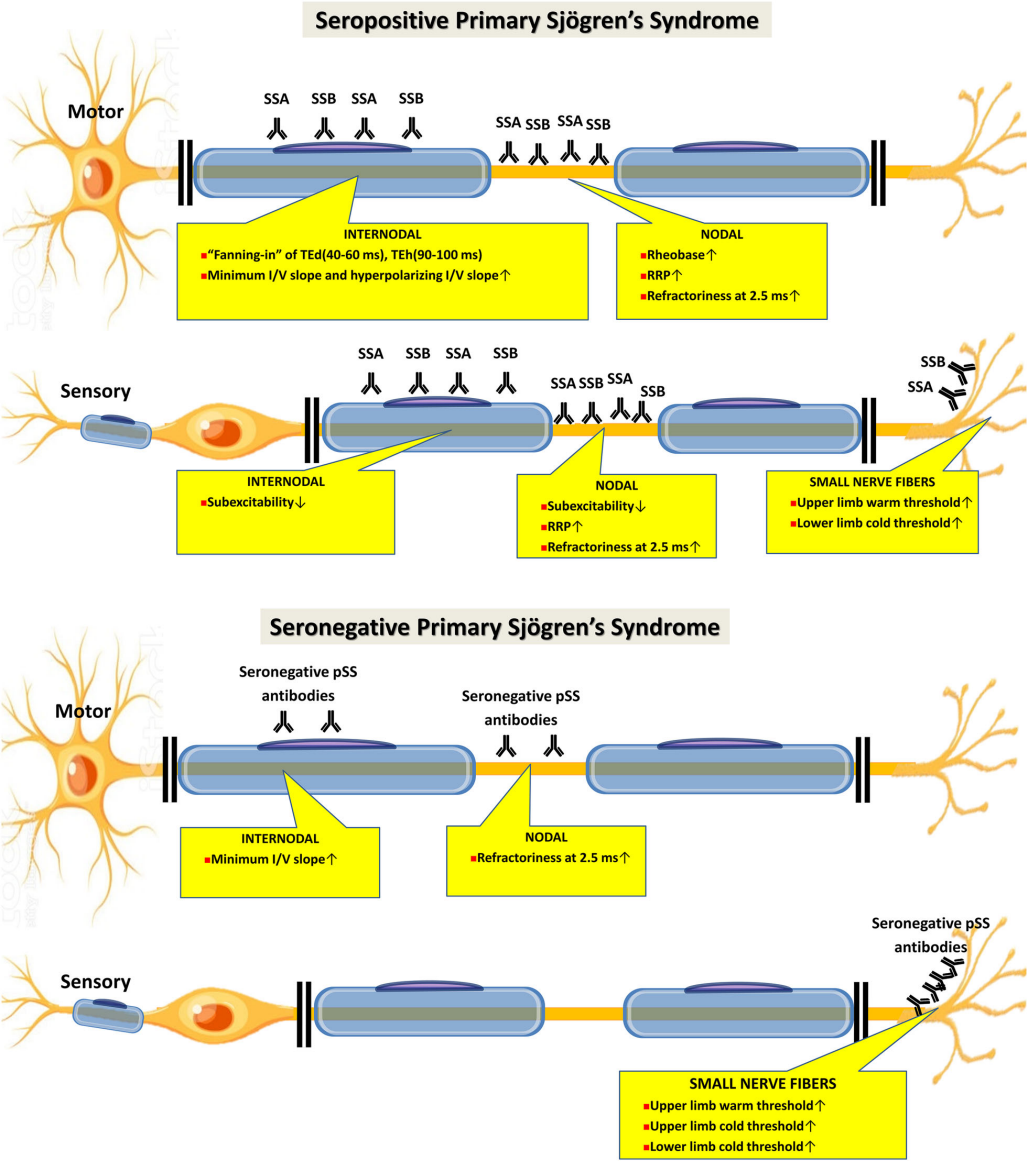
Axonal and small nerve fibers dysfunction in seropositive and seronegative pSS. In seropositive pSS, SSA, and SSB autoantibodies cause dysfunction in nodal and internodal region of the motor and sensory axon, and small nerve fibers. In seronegative pSS, the autoreactive antibodies cause dysfunction mainly in the small nerve fibers.

The present study revealed that the peripheral nervous system is affected differently in seropositive and seronegative pSS patients (Figure 3).

### Motor axonal dysfunction in seropositive pSS

In seropositive pSS, increased stimulus for 50% CMAP, increased rheobase, increased minimum I/V slope and hyperpolarizing I/V slope, increased RRP, and increased accommodation of TE toward hyperpolarizing current suggests that the motor axon is in a hyperexcitable state, and compatible with axonal depolarization.^22^

Prominent changes in rheobase, RRP, and refractoriness at 2.5 ms suggested dysfunction of nodal area. Meanwhile, prominent changes in TEd(40–60 ms) and TEh(90–100 ms), as well as minimum I/V slope and hyperpolarizing I/V slope suggested abnormality of the internodal region. The above findings might infer that the anti‐SSA and anti‐SSB antibodies exert its effects mainly on the nodal and internodal region.

Correlation between various motor axonal parameters between anti‐SSA level, anti‐SSB level, and duration of dry eyes, dry mouth, and neurological symptoms in pSS suggested that the motor nerve axonal study could detect a cumulative neuropathologic effect of the anti‐SSA and anti‐SSB autoantibodies. Nevertheless, the symptoms caused by motor axonal changes in the present study are subclinical, as motor symptoms observed in our patients are not prominent.

### Motor axonal dysfunction in seronegative pSS

Motor axonal study of seronegative pSS shown only a trend toward being depolarized, with increased minimum I/V slope and increased refractoriness at 2.5 ms. These changes suggested a less prominent nodal and internodal involvement in seronegative pSS.

### Sensory axonal dysfunction in seropositive pSS

Sensory axonal study of seropositive pSS shows increased stimulus for 50% SNAP, decreased latency, increased RRP, increased refractoriness at 2.5 ms, and increased subexcitability, with a trend of increased accommodation toward depolarizing and hyperpolarizing current, which is also compatible with depolarization. The changes in RRP and refractoriness at 2.5 ms localized the dysfunction to nodal region, while changes in subexcitability point to nodal and internodal abnormality.

Correlation between the above parameters with the duration of pSS symptoms (RRP and refractoriness at 2.5 ms) and with anti‐SSA/anti‐SSB level suggested that these axonal parameters also reflects cumulative axonal pathology of pSS and anti‐SSA/anti‐SSB antibodies.

A correlational relationship is also found between SF‐36 MCS with subexcitability and TEh(overshoot), meaning that the more severe dysfunction in these axonal parameters was correlated with worse life quality, especially in the mental component.

### Sensory axonal dysfunction in seronegative pSS

No significant sensory nerve excitability parameter changes in the sensory axonal study of seronegative pSS are observed, suggested that autoreactive antibodies might exert minimal effects in the sensory axon.

Previous studies have reported both axonal degeneration and myelinated fiber loss on nerve biopsy,^23^ dorsal root ganglionitis,^24^ and peripheral neuropathy with vasculitis and involvement of vasa vasorum in pSS patients.^25^, ^26^, ^27^ Axonal dysfunction observed in the present study is in accordance with the pathological findings with previous studies. Although anti‐SSA/SSB are a primarily anti‐nuclear antibody, it has been shown to have a direct role in damaging tissues.^28^ SSA and SSB antigens could be expressed on the cell surface in various circumstances.^28^, ^29^, ^30^ Thus, some of the possible explanations for axonal dysfunction causes by the autoantibodies are: (1) anti‐SSA/SSB exerts its neuropathic effect by causing vasculitis that affects vasa vasorum and peripheral nervous system, or (2) anti‐SSA/SSB causes axonal degeneration and myelinated fiber loss due to nodal/internodal expression of SSA/SSB antigens induced under certain circumstances.

### Symptomatic presentation, sensory axonal dysfunction, and small nerve fibers involvement in pSS

The proportion of patients suffering from the motor and sensory symptoms did not significantly differ between seropositive and seronegative pSS. Direct comparison of NPSI score and maximal pain score between seropositive and seronegative patients showed no statistically significant difference, and pain symptoms do not appear to be milder in seronegative patients.

In the present study, the thermal QST study does confirm the prominent dysfunction of small nerve fibers in both seronegative and seropositive pSS. As sensory nerve excitability study suggested that seronegative pSS has less prominent nodal and internodal sensory axonal dysfunction as compare to seropositive pSS, one possible explanation is that small nerve fibers involvement probably played an important role in causing neuropathic pain in seronegative pSS. The exact pathogenesis of pain‐inducing small nerve fibers dysfunction in pSS should be carefully explored in future studies.

## Conclusion

The present study suggests that in seropositive pSS, anti‐SSA and anti‐SSB autoantibodies cause dysfunction in nodal and internodal region of the axon and small nerve fibers; Meanwhile, in seronegative patients, the autoreactive antibodies probably exert its neuropathogenic effects mainly by affecting the small nerve fibers instead of the axon. Nevertheless, the clinical findings, TNSr, and the duration of neurological symptoms do not significantly differ between seropositive and seropositive patients.

Thus, the study reveals that although the neurological symptoms in seropositive and seronegative pSS are similar, the underlying pathophysiology for the two types of pSS is different.

## Author Contributions

J. Tani, C.S.Y. Lin, and J.Y. Sung, contributed to the study conception and design, data interpretation, drafting, and revision of the manuscript. J. Tani, H.T. Liao, H.C. Hsu, L.F. Chen, T.S. Chang, and J.Y. Sung participated in data acquisition. J. Tani and J.Y. Sung performed the statistical analysis.

## Conflict of Interest

None of the authors report any disclosures relevant to the manuscript.
